# Interfacial valence electron localization and the corrosion resistance of Al-SiC nanocomposite

**DOI:** 10.1038/srep18154

**Published:** 2015-12-15

**Authors:** Sareh Mosleh-Shirazi, Guomin Hua, Farshad Akhlaghi, Xianguo Yan, Dongyang Li

**Affiliations:** 1 Department of Chemical and Materials Engineering, University of Alberta, Edmonton, T6G 2V4, Alberta, Canada; 2 School of Metallurgy and Materials Engineering, University College of Engineering, University of Tehran, Tehran, 14174, Iran; 3 School of Mechanical Engineering, Taiyuan University of Science and Technology, Taiyuan, 030024, Shanxi, People’s Republic of China

## Abstract

Microstructural inhomogeneity generally deteriorates the corrosion resistance of materials due to the galvanic effect and interfacial issues. However, the situation may change for nanostructured materials. This article reports our studies on the corrosion behavior of SiC nanoparticle-reinforced Al6061 matrix composite. It was observed that the corrosion resistance of Al6061 increased when SiC nanoparticles were added. Overall electron work function (EWF) of the Al-SiC nanocomposite increased, along with an increase in the corrosion potential. The electron localization function of the Al-SiC nanocomposite was calculated and the results revealed that valence electrons were localized in the region of SiC-Al interface, resulting in an increase in the overall work function and thus building a higher barrier to hinder electrons in the nano-composite to participate in corrosion reactions.

Microstructural inhomogeneity generally deteriorates the corrosion resistance of materials due to the galvanic effect and interfacial issues. The difference in electrochemical behavior between different phases may accelerate corrosion reactions, leading to higher susceptibility to electrochemical attacks. For metal-matrix composites in which the reinforcing phases are usually non-conductive ceramic materials, the interfacial properties become crucial.

The interface between the matrix and reinforcement plays an important role in property control for composites^1^. As an example, Al-matrix composites (AMCs), possessing high strength-to-weight ratio, high thermal and electrical conductivities, are important materials for automotive, aerospace and civil applications^2^. However, one of main obstacles that limit the application of AMCs is related to the interface in AMCs, which often serve as a source for corrosion. The interface between the Al matrix and inclusions provides sites for corrosion to occur preferentially^3^,^4^. Pitting attack is often reported as a major form of corrosion in Al-SiC composites^5^,^6^. From the point of view that the corrosion is dominated by electron activation behaviour besides passive or transpassive properties^7^, electronic properties of interfaces such as polarizability and localization, which are related to the activity of valence electrons, are requisite to understand the corrosion behaviour of AMCs. In recent years, considerable attention has been paid to nanocomosites, in which the reinforcements are nano-sized, due to their superior mechanical properties^8^,^9^,^10^. For nano-composites in which the fraction of interfaces is increased by orders of magnitude with significantly reduced spacing between reinforcements, the activity of valence electrons in the system could be markedly affected. This is an issue worth being looked into in order to optimize the nano-composites.

In general, the chemical reactivity of materials could be evaluated by their polarizability, which is a property for measuring how the material responds to the external field^11^. Although the accurate polarizability is difficult to be calculated due to excited states are involved^12^,^13^, the polarizability is essentially determined by the valence electron density^14^,^15^. Moreover, the polarizability exhibits correlation with other optical and mechanical properties which are strongly affected by valence electron density^16^. In view of these evidences, a question is then arisen whether or not other parameters determined by valence electron density can serve with the capability similar to that of polarizability for studying chemical reactivity of materials.

Electron work function (EWF), as an important physical property to evaluate the driving force for the electron flow, has been widely applied in microelectronics^17^, field emission^18^, catalysis^19^ and solar cell^20^ etc. In our early studies, it has been demonstrated that EWF is determined by the valence electron density according to the image charge method^21^. Therefore, efforts were made to explore application of EWF on the chemical reactivity characterization. In this study, how the EWF and the corrosion resistance of Al-SiC nanocomposite changed with the interfacial electron state were investigated. The matrix is Al6061 and is termed here as Al matrix for simplicity. Along with the electron localization function (ELF) determined by first-principle calculation, the electronic origin responsible for the interfacial enhancement on the corrosion resistance of the material become understandable.

## Experiment and Calculation Details

### Materials and characterizations

SiC nanoparticles (25–50 nm, Plasma-Chem Co. Germany) were co-milled with nitrogen gas atomized Al-6061 powder (38–63 μm) in a laboratory planetary ball mill (PM-2400). The mixed powers contained 1, 2 and 3 vol% SiC nanoparticles, respectively. The nominal chemical composition of Al6061 is given in Table 1. The milling process proceeded in argon for 20 h and hardened stainless steel balls of 10 mm in diameter were used. The ball to powder weight ratio and rotational speed were 15:1 and 300 rpm, respectively, and 1.5 wt% of stearic acid (S.A.) was used as the Process Control Agent (PCA). The powder mixtures were then cold pressed in a steel die under a constant pressure of 750 MPa. Green compacts were hot extruded at 500 °C and held for 45 min using a 45-tonnes hydraulic press at a ram speed of 1 mm/s with the extrusion ratio of 25:8. For simplicity, we denote the fabricated as Al-SiC nanocomposite rather than Al6061-SiC nanocomposite. Microstructures of the nanocomposite samples were characterized using a scanning electron microscope (CamScan MV2300,UK) and Transmission Electron Microscope (a Hitachi H-7000). X-ray diffraction (XRD) was used to determine phases in the nanocomposites using a Philips X’Pert MPD diffractometer with Cu Kα radiation. Electron work functions (EWFs) of the samples were measured using a scanning Kelvin probe with a gold tip, provided by KP Technology (Caithness, UK). Before the test, each specimen was ground with silicon carbide papers up to 1200-grit, polished and cleaned with acetone.

The corrosion behavior of the samples respectively in 3% NaCl and 0.1 mol/l H_2_SO_4_ solutions was evaluated through electrochemical polarization measurement. Before the polarization test, each specimen connected to a copper wire was mounted in epoxy resin with a surface area of 1 cm^2^ exposed to the corrosive solution. The exposed surface was ground with silicon carbide papers up to 800-grit and cleaned with acetone. A saturated calomel electrode (SCE) was used as the reference electrode and a platinum plate with area of 1 cm^2^ was used as the counter electrode. The corrosion current density was determined using the Tafel extrapolation method.

### Computational calculation

The first-principle method was employed to calculate work function of (111) plane of Al and (100) plane of silicon carbide (SiC), and the electron localization function (ELF) of valence electrons in interfacial zone between Al and SiC. The interface was constructed by connecting (111) plane of Al with (100) plane of SiC, followed by relaxation of the system to minimize the lattice misfit energy. The calculations were implemented with an ABINIT package^22^,^23^. The Norm conserving pseudo-potentials^24^ and Perdew-Burke-Ernzerhof Generalized Gradient Approximation (GGA) of exchange-correlation functional^25^ were adopted for the calculation. An energy cut-off of 36 Hartree (1Hartree = 27.211 eV) and a 6 × 6 × 1 k-point mesh was used to achieve self-consist field convergence with the tolerant potential V(r) residual less than 10^−8^ Hartree. As for the ELF, it is defined as^26^,^27^


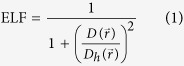


where


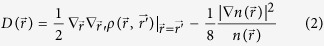


and


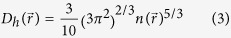


where *ρ* is the first-order reduced density matrix with spin dependence, 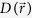 is the kinetic energy functional, 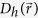 is the kinetic energy density of uniform electron gas with a spin density equal to the local 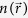. The equations indicate that the value of ELF is close to the unity when valence electrons are highly localized, while it decreases to 0.5 when the distribution of valence electrons is similar to that of the homogeneous electron gas.

## Results and Discussions

**Figure 1:**
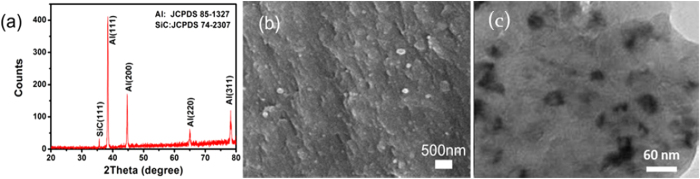
(**a**) XRD pattern, (**b**) SEM image, and (**c**) TEM image of Al-3%SiC nanocomposite.

In Fig. 1, X-ray diffraction pattern (XRD), typical SEM and TEM images of the Al-3%SiC nanocomposite are presented. The prepared Al-3%SiC nanocomposite consisted of FCC structured Al matrix (Al6061) and Cubic structured SiC inclusions. No other phases, such as undesired Al_3_C_4_, were observed in the nanocomposite. From the SEM image, one may see that SiC nanoparticles were embedded in the Al matrix in a satisfied manner, no aggregation of SiC nanoparticles was observed on the surface. Furthermore, the TEM image also shows that the SiC nanoparticles are homogeneously distributed in the Al mixture and the particle size ranges from 20 nm to 60 nm.

**Figure 2:**
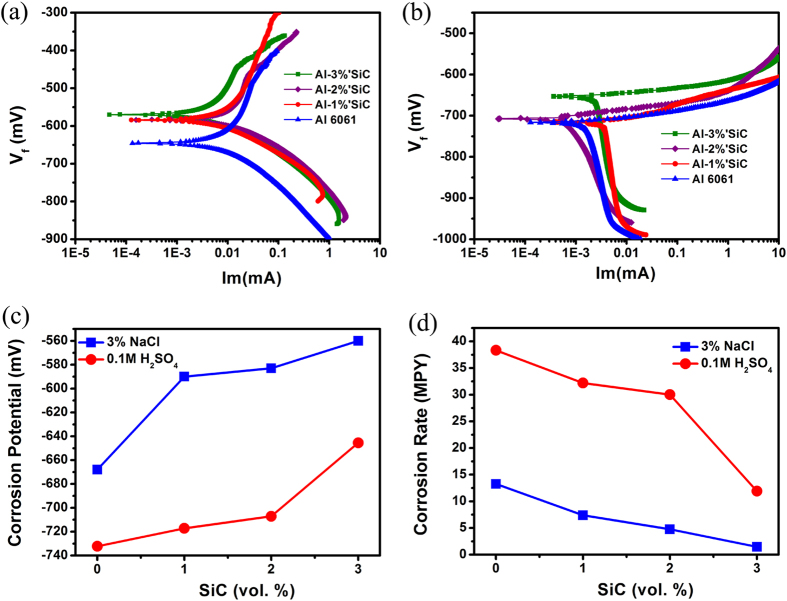
Polarization curves of Al-SiC nanocomposites in (**a**) 3%NaCl and (**b**) 0.1M H_2_SO_4_; (**c**) Corrosion potential versus the volume fraction of SiC nanoparticles in two corrosive solutions, respectively, and (**d**) Corrosion rate versus the volume fraction of SiC nanoparticles in the two corrosive solutions, respectively.

Polarization curves, corrosion potential and corrosion rates of Al-SiC nanocomposites with different volume fractions of SiC nanoparticles in 3 wt.% NaCl and 0.1M H_2_SO_4_ solutions, respectively, are presented in Fig. 2. The H_2_SO_4_ solution is a strong corrosive medium and can corrode various metals in a uniform corrosion mode. In contrast, in the NaCl solution, Cl^−^ ions play a main role in corrosion, usually resulting in pitting and intergranular corrosion^28^. In both the solutions, corrosion rate of the nanocomposite decreased with increasing the amount of SiC nanoparticles as shown in Fig. 2(d). It was noticeable that the added SiC nanoparticles did not deteriorate the corrosion resistance of Al-SiC nanocomposite due to any possible galvanic effect or lower interfacial resistance to corrosion attacks. Instead, the corrosion resistance was considerably improved.

The damage to a material by corrosion is affected by two determining factors, one is corrosion rate that is related to corrosion kinetics, and another is the corrosion tendency or potential. The lowered corrosion rate of Al6061 with SiC nanoparticles, compared to that of Al6061, could be partially ascribed to the fact that the SiC particulates basically remained inert in the corrosive solutions, which would not promote local galvanic effect, and the dispersed SiC particles reduced the metallic surface area that was exposed to the solution, thus leading to lowered corrosion rate.

Another factor is the corrosion tendency, which is represented by the corrosion potential. A higher corrosion potential represents a lower tendency for corrosion. As shown in Fig. 2(c), in both corrosion solutions, the corrosion potential of Al6061 was raised by the added SiC nanoparticles, suggesting that adding SiC nanoparticles to the Al matrix reduced the driving force for corrosion. Such beneficial effect was enhanced by increasing the volume fraction of SiC nanoparticles as observed.

**Figure 3:**
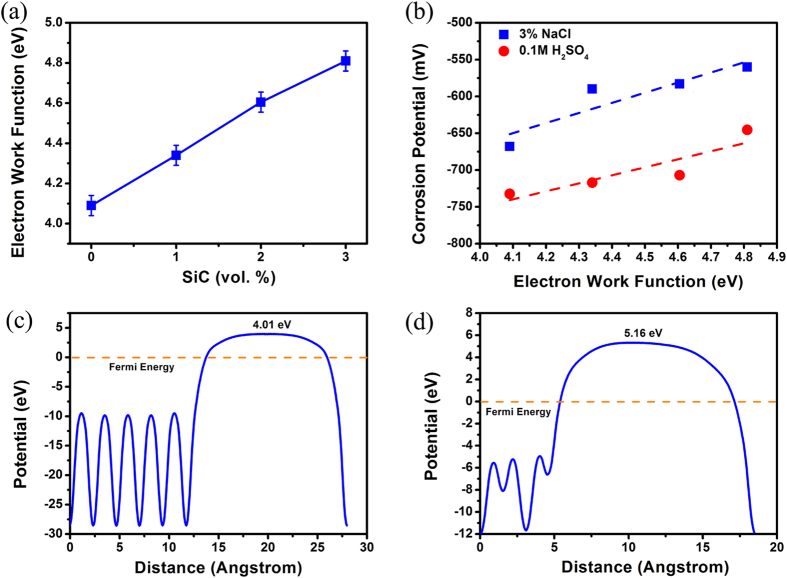
(**a**) EWFs of Al-SiC samples with different amounts of SiC nanoparticle, (**b**) observed linear relationship between the corrosion potential and EWF, (**c**) calculated electron work function of (111) plane of Al, and (**d**) calculated electron work functions of (100) plane of SiC.

The increase in the corrosion potential generally results from lowered activity of valence electrons that participate in corrosion reactions, which is reflected by raised electron work function (EWF). In order to see whether or not the EWF had the capability of monitoring the variation in activity of valence electrons with the interface density in nanocomposites, EWFs of the nanocomposites was measured and are illustrated in Fig. 3(a). As shown, EWF of the nanocomposite increased from 4.1 eV to 4.8 eV with an increase in the volume fraction of SiC nanoparticles. The intrinsic resistance of a material to corrosion is related to its electron behavior or the work function while its corrosion potential or electrochemical potential is environment-dependent. The relationship between the electrochemical potential (*ϕ*_*m*_) and the electron work function (*φ*_*m*_) of the metal can be generally expressed as^29^:


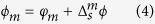


where 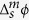 is the contact potential difference at the metal/solution interface. Thus, in different solutions, *ϕ*_*m*_ of a metal varies due to different values of 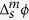 in different solutions, such as the salty and acidic solutions used in the present study. For the SiC/Al nanocomposites having different volume fractions of the same type metallic matrix, 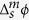 should be a constant. As a result, the corrosion potential and work function should be linearly correlated, which has indeed been confirmed as Fig. 3(b) illustrates.

In order to understand the mechanism for the increase in the overall EWF with the added SiC nanoparticles, we calculated electron work functions of Al (111) crystal plane and SiC (100) plane. Selecting these planes for the calculation is attributed to the fact that the low-index planes with low surface energies are thermodynamically stable and more representative. The calculation shows that electron work functions of Al (111) crystal plane and SiC (100) plane are 4.01 ev and 5.16 eV, respectively (see Fig. 3(c,d)). When a metallic element with a higher work function is added to another metal having a low work function, the overall work function can be increased 30, due to the fact that the former brings in more valence electrons, leading to an increase in valence or free electron density and consequently the work function. When a ceramic compound having a higher EWF is added to a metal matrix, an increase in the overall EWF is also expected. This has been proven by the experimental result shown in Fig. 3(a); however, the mechanism responsible for the increase in work function needs to be clarified, which has been done in the following paragraphs.

Considering the rise in EWF is an indication of an increase in valence electron density^21^, to which the polarizability is inverse proportional^15^, it is suggested that the increase in EWF may lead to a decrease in the polarizability or increased polarization resistance. Furthermore, owing to the decrease in polarizability, the material becomes more resistant to external fields and thus more inert^11^. It is therefore expected that lower polarizability corresponds to decreased chemical reactivity. Or in other words, the smaller the polarizability, the greater the chemical hardness^31^. As a result, the increase in EWF responds to a reduction of the chemical reactivity. The EWF appears to be a good indicator for the chemical reactivity of materials.

**Figure 4:**
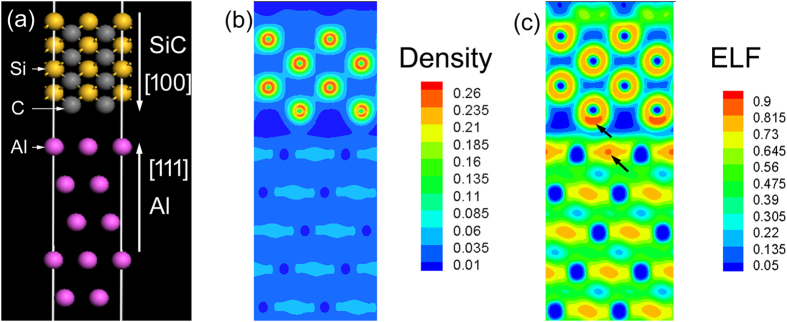
The atomic configuration at the Al-SiC interface (the grey atom is C, the yellow atom is Si, and the purple atom is Al), (**b**) the distribution of valence electron density, (**c**) ELF of valence electrons. As pointed by arrows, valence electrons in the interfacial region show higher localization, compared to those apart away from the interface.

An important issue here that needs to be clarified is “by which means the SiC nanoparticles can increase the EWF of the nanocomposites”. For a metallic material, its overall EWF is usually increased by adding elements with higher EWF if the added elements are dissolved in the material without forming second phases^30^. This happens because the density of valence electrons is increased when the higher-EWF elements are added. However, SiC is a non-metallic material. Considering a semiconductor-metal junction, where an electrical field could be built within the interface region under equilibrium condition^32^, the property of valence electrons in the vicinity of Al-SiC interfaces should be analyzed. In order to understand why the SiC nanoparticles raised the overall EWF, the electron density distribution and electron localization function (ELF) in the region of SiC-Al interface were calculated. The Al-SiC interface was constructed by connection between (111) plane of Al and (100) plane of SiC as illustrated in Fig. 4(a), since this configuration has maximal cohesive energy according to the study conducted by Luo *et al.*^33^. Calculated profiles of valence electron density distribution and ELF are presented in Fig. 4(b,c). From the valence electron density distribution, one may see that the valance electrons in carbon atoms are dragged downwards the interfacial region. This is consistent with the observation that interaction between Al and C atoms or the Al-C atomic bond is stronger than the Al-Si atomic bond^34^. The increase in EWF by adding SiC nanoparticles could be attributed to an increase in the electron density originating from the injection of electron from carbon atom electrons. Furthermore, according to the profile of ELF shown in Fig. 4(c), the electrons in the interfacial region have a higher degree of localization, as pointed by arrows, compared to those apart away from the interface no matter they are in the Al lattice or in the SiC lattice. This indicates that valence electrons are more confined in the interfacial region. With an increase in interfacial area as SiC nanoparticles added in Al matrix along with markedly reduced spacing between adjacent SiC nanoparticles, the confinement would be considerably enhanced to reduce the electron activity. This also resulted in a higher barrier to the escape of electrons from the system, corresponding to the raised electron work function. As a result, the Al-SiC nanocomposite possesses elevated corrosion resistance.

In summary, the corrosion behavior of SiC nanoparticle-reinforced Al6061 matrix composite in 3% NaCl and 0.1M H_2_SO_4_ solutions, respectively, were investigated. It was observed that the corrosion resistance of the Al-SiC nanocomposite was higher than the Al matrix (Al6061). Corresponding electron work function (EWF) of the Al-SiC nanocomposite increased, accompanied with an increase in the corrosion potential. The first-principle calculation indicates that the improvement in corrosion resistance is attributed to the electron localization in the interfacial region between the Al matrix and SiC particle. Such an effect would become profound when the SiC particle size is reduced to the nanoscale, with which the interfacial area is significantly increased along with markedly reduced spacing between adjacent SiC nanopartiles, leading to considerably enhanced electron confinement.

## Additional Information

**How to cite this article**: Mosleh-Shirazi, S. *et al.* Interfacial valence electron localization and the corrosion resistance of Al-SiC nanocomposite. *Sci. Rep.* **5**, 18154; doi: 10.1038/srep18154 (2015).

## Figures and Tables

**Table 1:** Chemical composition (wt %) of 6061 Aluminum alloy

**Mg**	**Si**	**Fe**	**Cu**	**Cr**	**Al**
1.12	0.64	0.48	0.33	0.04	Balance

## References

[b1] Hull D. An introduction to composite materials. (Cambridge University Press, Cambridge; New York, 1981).

[b2] Mobasherpour I., Tofigh A. A. & Ebrahimi M. Effect of nano-size Al2O3 reinforcement on the mechanical behavior of synthesis 7075 aluminum alloy composites by mechanical alloying. Materials Chemistry and Physics 138(2–3), 535–541 (2013).

[b3] Fu N. *et al.* In situ investigation of local corrosion at interphase boundary under an electrochemical-atomic force microscope. J Solid State Electrochem 1–8, 337–344 (2014).

[b4] Mishra A. K., Balasubramaniam R. & Tiwari S. Corrosion inhibition of 6061‐SiC by rare earth chlorides. Anti-Corrosion Methods and Materials 54 (1), 37–46 (2007).

[b5] Buarzaiga M. M. & Thorpe S. J. Corrosion Behavior of As-Cast, Silicon Carbide Particulate-Aluminum Alloy Metal-Matrix Composites. Corrosion 50 (3), 176–185 (1994).

[b6] Abdoli H. *et al.* Processing and surface properties of Al–AlN composites produced from nanostructured milled powders. Journal of Alloys and Compounds 490 (1–2), 624–630 (2010).

[b7] Callister W. D. Materials science and engineering: an introduction. (Wiley, New York, 2000).

[b8] Suryanarayana C. & Al-Aqeeli N. Mechanically alloyed nanocomposites. Progress in Materials Science 58 (4), 383–502 (2013).

[b9] Moazami-Goudarzi M. & Akhlaghi F. Effect of nanosized SiC particles addition to CP Al and Al–Mg powders on their compaction behavior. Powder Technol 245 (0), 126–133 (2013).

[b10] Ravindran P. *et al.* Application of factorial techniques to study the wear of Al hybrid composites with graphite addition. Mater Design 39 (0), 42–54 (2012).

[b11] Sabirov D. S. Polarizability as a landmark property for fullerene chemistry and materials science. Rsc Adv 4 (85), 44996–45028 (2014).

[b12] Mitroy J., Safronova M. S. & Clark C. W. Theory and applications of atomic and ionic polarizabilities. J Phys B-at Mol Opt 43 (20), 202001 (2010).

[b13] Kolandaivel P., Mahalingam T. & Sugandhi K. Polarizability and chemical hardness - A combined study of wave function and density functional theory approach. Int J Quantum Chem 86 (4), 368–375 (2002).

[b14] Bartolotti L. J. Polarizability as a Local Functional of the Electron-Density. P Indian as-Chem Sci 106 (2), 103–110 (1994).

[b15] March N. H. & Tosi M. P. Inverse relation between polarizability and valence-electron density at the nucleus. Journal of Molecular Structure: THEOCHEM 343 (0), 199–201 (1995).

[b16] Gilman J. J. Chemical and physical “hardness”. Mater Res Innov 1 (2), 71–76 (1997).

[b17] Zhou Y. H. *et al.* A Universal Method to Produce Low-Work Function Electrodes for Organic Electronics. Science 336 (6079), 327–332 (2012). 22517855 10.1126/science.1218829

[b18] You J. B. *et al.* Enhancement of field emission of the ZnO film by the reduced work function and the increased conductivity via hydrogen plasma treatment. Appl Phys Lett 94 (26), 262105 (2009).

[b19] Zasada F. *et al.* Potassium Promotion of Cobalt Spinel Catalyst for N(2)O Decomposition-Accounted by Work Function Measurements and DFT Modelling. Catal Lett 127 (1–2), 126–131 (2009).

[b20] Heo S. W., Lee E. J., Seong K. W. & Moon D. K. Enhanced stability in polymer solar cells by controlling the electrode work function via modification of indium tin oxide. Sol Energ Mat Sol C 115, 123–128 (2013).

[b21] Hua G. M. & Li D. Y. Generic relation between the electron work function and Young’s modulus of metals. Appl Phys Lett 99 (4), 041907 (2011).

[b22] Gonze X. *et al.* A brief introduction to the ABINIT software package. Z Kristallogr 220 (5–6), 558–562 (2005).

[b23] Gonze X. *et al.* ABINIT: First-principles approach to material and nanosystem properties. Comput Phys Commun 180 (12), 2582–2615 (2009).

[b24] Fuchs M. & Scheffler M. Ab initio pseudopotentials for electronic structure calculations of poly-atomic systems using density-functional theory. Comput Phys Commun 119 (1), 67–98 (1999).

[b25] Perdew J. P., Burke K. & Ernzerhof M. Generalized gradient approximation made simple. Physical Review Letters 77 (18), 3865–3868 (1996). 10062328 10.1103/PhysRevLett.77.3865

[b26] De Santis L. & Resta R. Electron localization at metal surfaces. Surface Science 450 (1–2), 126–132 (2000).

[b27] Savin A., Nesper R., Wengert S. & Fassler T. F. ELF: The electron localization function. Angewandte Chemie-International Edition in English 36 (17), 1809–1832 (1997).

[b28] Song G. L. & Haddad D. The topography of magnetron sputter-deposited Mg–Ti alloy thin films. Materials Chemistry and Physics 125 (3), 548–552 (2011).

[b29] John O’. M. B. & Shahed U. M. K. Surface electrochemistry: a molecular level approach. (Springer, New York, 1993).

[b30] Lu H., Hua G. & Li D. Dependence of the mechanical behavior of alloys on their electron work function—An alternative parameter for materials design. Appl Phys Lett 103 (26), 261902 (2013).

[b31] Gilman J. J. Electronic Basis of the Strength of Materials. (Cambridge University Press, 2003).

[b32] Li S. S. Semiconductor physical electronics. (Plenum Press, New York, 1993).

[b33] Luo X., Qian G. F., Wang E. G. & Chen C. F. Molecular-dynamics simulation of Al/SiC interface structures. Phys Rev B 59 (15), 10125–10131 (1999).

[b34] Wenchang L., Kaiming Z. & Xide X. Adsorption of aluminum on β-SiC(100) surfaces. Phys Rev B 45 (19), 11048–11053 (1992). 10.1103/physrevb.45.11048 10001027

